# Supplementary material for: Attenuation Imaging with Ultrasound as a Novel Evaluation Method for Liver Steatosis
Source: J Clin Med. 2021 Mar 2;10(5):965. doi: 10.3390/jcm10050965 (PMC7957732; doi:10.3390/jcm10050965)
Supplement: Supplementary File 1 [file jcm-10-00965-s001.pdf]

The mechanism and operation method of attenuation imaging

ATI is determined by data obtained using TOSHIBA®i800 (Toshiba, Tokyo, Japan) ultrasound equipment and is manipulated by technicians who are not aware of the re-sults of other reports.

ATI protocol

(1) At least 5 valid data points will be collected.

(2) The success rate is over 60%.

(3) Every R2 value is 0.9 or greater, and data points are recorded. (4) The interquartile range is less than 30% of the median ATI.

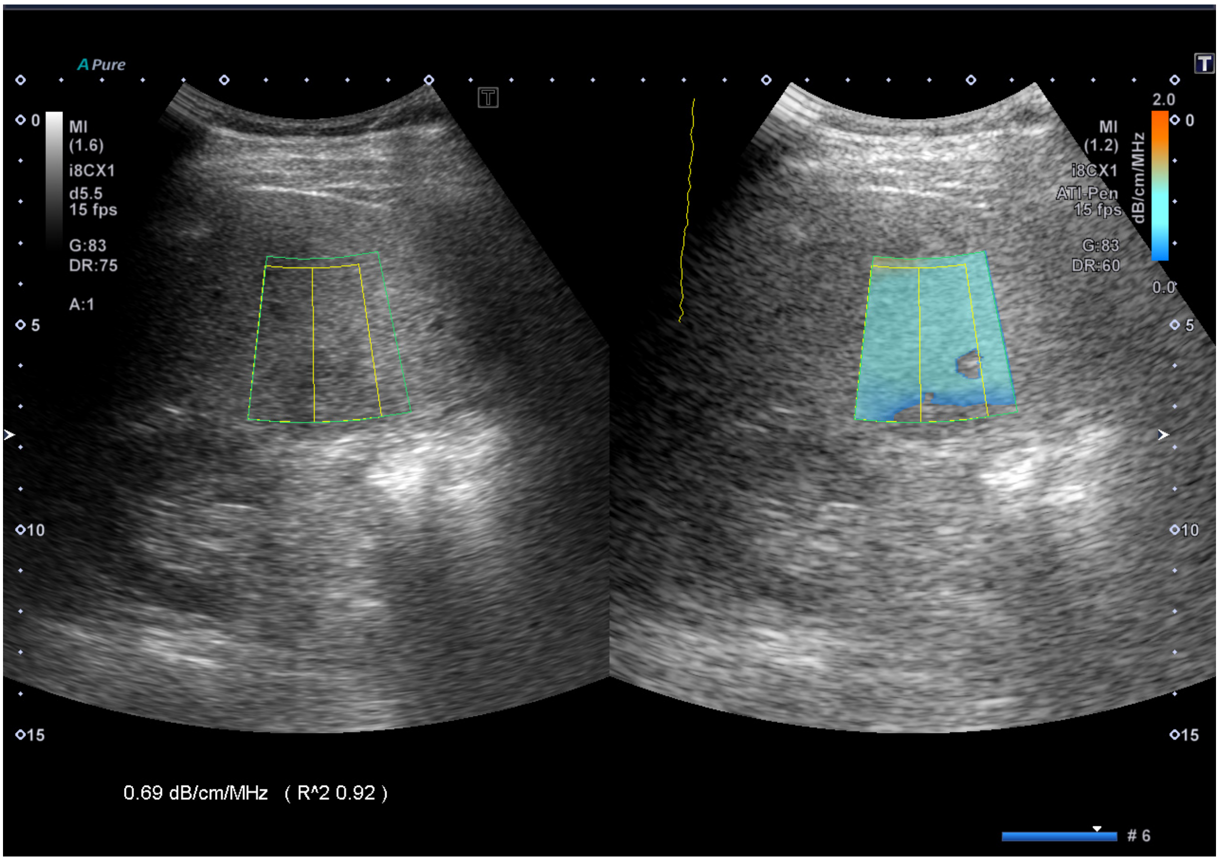

Figure S2. Case 1: biospy-proven steatosis grade 0 with attenuation imaging 69 db/mm/MHz

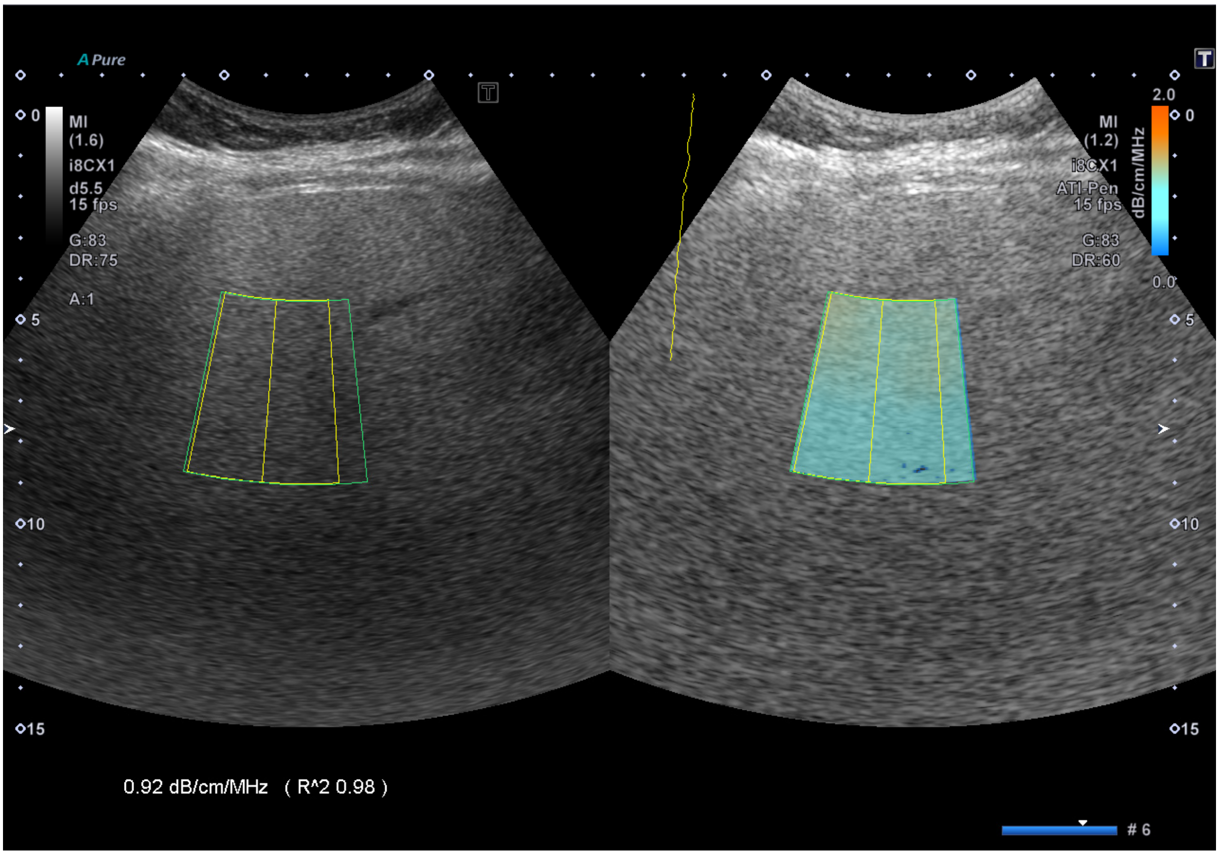

Figure S3. Case 2: biospy-proven steatosis grade 2 with attenuation imaging 92 db/mm/MHz

All the related knowledge of attenuation imaging with TOSHIBA®i800 (Toshiba, Tokyo, Japan) is in this link:

https://1drv.ms/p/s!Auav2ZlZkZSmpGTnNRDMklN9vwnd

And also in youtube:

https://youtu.be/7Fw9msBRvnw
